# Time flies, but you’re in control: the mediating effect of self-control between time attitude and academic procrastination

**DOI:** 10.1186/s40359-023-01438-2

**Published:** 2023-11-13

**Authors:** Juanjuan Wang, Yi Sun

**Affiliations:** 1 School of English Teacher Education, Xi’an International Studies University, Xi’an, China; 2 Linguistics and Applied Linguistics Research Center, Guangdong University of Foreign Studies, Guangzhou, China

**Keywords:** Academic procrastination, Time attitude, Self-control, Chinese middle school students

## Abstract

**Background:**

Academic procrastination has become an increasing concern in the educational sector. Prior studies identified various correlations among academic procrastination, time attitude and self-control. Nevertheless, few studies have examined the past time attitude and the mechanism underlying those relationships, and the existing studies have been implemented during regular school time. To fill those gaps, the present study includes all three dimensions of time attitude (past-oriented, present-oriented and future-oriented in both positive and negative perspectives), and proposes self-control as a mediator between academic procrastination and time attitude. The study was carried out during an extraordinary winter holiday in China, with final exams waiting at the end of the holiday.

**Methods:**

A total of 323 middle school students in China (178 girls and 145 boys, 12–19 years old) completed an online survey with questions on their academic procrastination, time attitude and self-control. The collected data were analyzed using IBM SPSS Statistics 25 and Hayes SPSS macro PROCESS (Model 4).

**Results:**

The results showed that academic procrastination was negatively associated with all three positive time attitudes and positively correlated with the present negative time attitude. Moreover, self-control significantly mediates the relationship between academic procrastination and all three positive time attitudes.

**Conclusion:**

Based on these findings, self-control could be the underlying mechanism in the relationship between academic procrastination and time attitude. This study broadens the scope of relevant empirical research to the past time attitude, and determines the mechanism that underlies the association between academic procrastination and time attitude under a novel context. Further implications for teaching regulation and intervention are discussed.

**Supplementary Information:**

The online version contains supplementary material available at 10.1186/s40359-023-01438-2.

## Introduction

A holiday is a period of rest from places such as work and school [1]. However, Chinese junior and senior middle school students had an extraordinary holiday last winter. Because the nationwide lockdown was lifted on 6 December 2022 in China, nearly all middle schools did not hold final exams at the end of the autumn semester, and postponed them until the beginning of the following spring semester. Therefore, students were faced with preparing for their final exams during the winter holiday. Studies have acknowledged that preparing for exams is a crucial phase for learning, but have also reported procrastination tendencies during this phase [2]. With the onset of COVID-19, academic procrastination has become an increasing concern in the educational sector [3]. Under this unprecedented situation, would Chinese middle school students fully utilize this period to prepare for the coming exams, or irrationally procrastinate and even avoid the review activities to enjoy their holiday instead? [4] This study aims to investigate their tendency to procrastinate during this special winter holiday.

Procrastination is “a temporal self-regulation failure that reflects a disjunction between the present and future self” [5]. This suggests that time attitude is a significant contributing factor to procrastination. A present-oriented time attitude increases the likelihood of procrastination, while a future-oriented time attitude decreases the risk of it [6]. However, the existing studies have not taken the past time attitude into consideration [7], even though it is also believed to be related to procrastination [8]. Therefore, this study intends to further this topic under this extraordinary context by expanding time attitude to all three dimensions (past-oriented, present-oriented and future-oriented).

Moreover, time attitude is correlated with self-control [9]. Self-regulation Theory [10] proposes that self-control influences the link between traits and behavioural outcomes [11]. Thus, we expect a mediating effect of self-control between time attitude, a cognitive-psychological trait, and academic procrastination, a failed self-regulation behaviour. However, there is little empirical research to investigate the mediating effect under this novel situation, especially on Chinese middle school students.

Taken together, the existing studies can be furthered by including more dimensions of time attitude, and tackling the underlying mechanism in the relationship between academic procrastination and time attitude. Thus, the present study will attempt to include these by examining Chinese middle school students during the novel winter holiday. The specific research questions for this study are: Is academic procrastination associated with all dimensions of time attitudes? Does self-control mediate the association between academic procrastination and all three time attitudes among Chinese middle school students in the extraordinary winter holiday?

The present study contributes to the field in the following ways. First, most of them use ZTPI (Zimbardo Time Perspective Inventory) [12] to measure time perspective, and merely focus on the relationship between future-oriented time attitude and procrastination. Instead, ATAS (Adolescent Time Attitude Scale) [13] is more suitable for middle school students, and covers all three dimensions of time attitude [14]. Thus, this study employs ATAS to investigate the correlation between all three dimensions of time attitude and procrastination. Second, all of them were carried out during regular school time. The unprecedented winter holiday, with exams waiting at its end, is expected to influence students’ time attitude and holiday activities. Whether they can resist the temptations to enjoy the holiday and make full preparations for the exams depends heavily on their self-control. Third, there are only two studies on this topic [6, 7]. Kim et al.’s study identified the relationship between time attitude, self-control and academic procrastination, but they recruited Korean college students as participants [6]. Yet, academic procrastination has been found to permeate among middle school students. For example, 39.5% of Chinese middle school students reported a tendency to procrastinate [15]. Regarding the detrimental effect of procrastination on students’ academic performance and mental well-being [16], it’s high time to investigate the mechanism underlying procrastination among middle school students. The other study by Li and Lv determined the mediating role of achievement motivation other than self-control [7]. To deepen our understanding of procrastination and take into account its complex nature, this study suggests that self-control can act as a mediator.

The article is organized as follows: Sect. 2 introduces related studies to lay the foundation for the research hypothesis; Sect. 3 reports the details of the research method, including participants’ information, measures, data collection and procedure, and data analysis; Sect. 4 describes the results; Sect. 5 is an overall discussion of the findings; and finally, Sects. 6 to 9 present implications, limitations, further perspectives, as well as a conclusion.

## Related studies

### Procrastination

Procrastination is the tendency to postpone or completely avoid an activity under one’s control [17]. It results from a combination of factors, such as doubt of one’s ability to perform a task, inability to delay gratification, and blame for one’s difficult situation on external sources [18, 19]. Academic procrastination refers to this irrational behaviour in learning activities. Learners tend to postpone important and necessary learning tasks regardless of possibly being worse off [20], suggesting a disjunction between the present and future self [5]. As time passes, this failure of temporal self-regulation leads to a decline in academic performance [21, 22]. Therefore, it is necessary to predict students’ procrastination tendencies, in order to assist those who may have such tendencies in overcoming it, or to provide instructors with timely interventions [17, 23].

Procrastination is associated with various factors. First, a growing number of studies indicate that procrastination is related to negative feelings, such as high levels of stress [24], frustration and self-blame [6]. These negative thoughts lead to a preoccupation with personal flaws that contribute to a lack of concern for the future, because they focus more on the pleasant present to avoid challenging tasks and their possible negative consequences [5]. Second, procrastination has also been found to be caused by an emphasis on the present as opposed to the future [6]. For this intrinsic time trait of procrastination, researchers have attempted to identify the relationship between procrastination and people’s time perspectives [25]. Generally speaking, procrastination is positively associated with the future time perspective, and is less consistently associated with the present time perspective. Some previous studies detected a positive association between procrastination and present-hedonistic time orientation [6, 26–28], while others failed to find this association [29, 30]. The inconsistent findings highlight the need for additional research on the topic. Besides, as aforementioned, the prior studies paid little attention to the relationship between procrastination and past time attitude. Yet there are three time perspectives: the past, the present, and the future [31]. Further investigation into all three dimensions may reveal a more comprehensive relationship between these two closely-linked variables. Lastly, the extraordinary winter holiday with exams waiting at the end can offer additional evidence for the relationship between procrastination and time perspective under more diverse contexts.

### Time attitude

Time perspective is one’s attitude to the passage of time [12]. It consists of the past time perspective, the present time perspective and the future time perspective. The past and present time perspectives are divided into positive and negative dimensions, whereas the future time perspective has no subdivision. Later, Carelli et al. [32] also divided the future time perspective into positive and negative dimensions. Hence, there are altogether six pairs of positive and negative time perspectives. Time attitude is the emotional aspect of time perspective concerned with adolescents’ attitude or emotional experiences toward the past, the present and the future [31]. It also consists of six dimensions, in accordance with that of the time perspective. It is more applicable to adolescents [14, 33], and thus will be our focus in this study.

Previous studies indicated that time attitude is significantly associated with students’ academic procrastination [6–8]. On the one hand, academic procrastination positively correlates with a negative time attitude. For example, learners might think: “No matter what, I will fail in the future (future negative attitude), so why should I bother to do it now [20]?” Or “past experiences prove that I cannot do it well, and I cannot do it well now, either” (present negative attitudes) [8]. On the other hand, academic procrastination negatively correlates with a positive time attitude, because people with a positive time attitude also have high esteem, self-efficacy and well-being [31, 34], which all contribute to timely and effective actions. However, most existing studies have employed ZTPI rather than ATAS, which is more suitable for measuring adolescents’ time attitude. Besides, they paid little attention to past time attitude, which is also believed to correlate with procrastination [8]. The requirement of a more suitable measurement of time attitude and the ignorance of the past time attitude both suggest the necessity to further investigate this topic. In addition, few existing studies enrolled Chinese middle school students as participants. This study thus uses ATAS to investigate Chinese middle school students’ time attitude in all three dimensions during this special winter holiday.

### Self-control

Self-control is the capability to control one’s own cognition, emotions and behaviours by delaying immediate gratification and adjusting to situational and social needs without external enforcers [6]. It is one of the two main processes within the self-regulatory framework in social sciences [11]. Self-regulation is the process of exerting control over oneself to align with a desired standard [10]. It operates through two processes: monitoring, which determines how far off a goal the current progress is, and self-control, which involves resisting the urge to change or maintain behaviour. An individual with high self-control is advantageous in situations with unambiguous standards, such as academic behaviours [11].

Studies have noted the close link between self-control and academic procrastination [21]. On the one hand, poor self-control leads to procrastination [35, 36]. Students with low self-control have difficulty with learning procedures, and are not good at balancing the speed of performing a task with the time of completion. All of these result in academic procrastination [37, 38]. On the other hand, students with high self-control can properly use strategies to adjust learning behaviours, thus successfully preventing procrastination [38]. At the same time, self-control has also been found to be related to time attitude. Future time attitude is positively correlated with a high level of self-control [9].

In a nutshell, prior studies have identified relationships among time attitude, academic procrastination and self-control. This reminds us of the previous research on self-control as a mechanism linking traits and behaviour outcomes [11]. Specifically, time attitude, as a cognitive-psychological trait [8], may regulate procrastination behaviour through the mediating effect of self-control. Yet, there are only two empirical investigations into this issue: Kim et al. [6] and Li and Lv [7]. Kim et al. [6]’s results showed that a present-oriented time perspective brings low self-control, which increases the likelihood of procrastination, while a future-oriented time perspective leads to high self-control, which reduces the tendency of procrastination. This study merely focused on present and future time attitude, and had Korean college students as participants. Li and Lv [7]’s study testified the mediating effect of achievement motivation other than self-control among Chinese middle school students during regular school time. Therefore, this study aims to provide more diversified evidence for the topic among Chinese middle school students during this novel winter holiday. We hypothesize that: (1) academic procrastination is negatively correlated with all three positive time attitudes (past, present, and future), and is positively associated with all three negative time attitudes (past, present and future); (2) self-control may mediate the relationship between academic procrastination and all six time attitudes among Chinese middle school students.

## Method

### Participants

We applied a survey method and recruited questionnaire participants online. To obtain a representative sample, we used the sample service provided by the Questionnaire Star platform, an online questionnaire service website. The platform distributed the questionnaire link to potential middle school students or their parents through WeChat or E-mail. All students voluntarily participated in the survey and gave their informed consent. A small amount of money as a reward was available through digital red envelopes after they finished all the questions. A total of 326 (179 girls and 147 boys; 12–19 years old) junior (176) and senior (150) middle school students, all Chinese native speakers from 13 provinces and regions in China, took part in our online survey. This sample size meets the guidelines for exploratory research during the COVID-19 pandemic [39].

### Measures (see Appendix)

**Academic Procrastination Questionnaire for Chinese middle school students (APQC)**.

APQC (in the Chinese language) [40] was adapted from The Procrastination Scale (TPS) developed by Tuckman [4]. TPS contains the fewest items (16) compared with other measures. For example, there are 44 items in the Procrastination Assessment Scale-Students by Solomon and Rothblum (1984). The small number of questions aims to reduce participants’ stress when finishing the questionnaire. More importantly, the Chinese version of TPS shows high reliability among both Chinese middle school students (α = 0.90) [7] and college students (α = 0.81) [41]. We thus adopted its Chinese version for Chinese middle school students (APQC)(α = 0.79) [40]. APQC consists of 20 items, covering homework procrastination, self-study procrastination, and exam-preparation procrastination. For example, participants might read such a question: *I wait till the last minute to begin doing homework*. Each item is rated on a five-point Likert scale ranging from “never” (1) to “always” (5). The higher the score, the more serious the procrastination. In the present study, its Cronbach α is 0.87, and KMO value is 0.66.

### Chinese version of the adolescent time attitude scale (CATAS)

CATAS is the Chinese version of the Adolescent Time Attitude Scale [13]. ATAS is designed for adolescents attending junior and senior middle schools. Our participants all belong to this age group. Besides, its Chinese version displays high reliability in Chinese high school and college students, ranging from 0.77 to 0.86 [33]. CATAS consists of 30 items with five items for each dimension of time attitude: past positive, past negative, present positive, present negative, future positive, and future negative. For example, participants might read such a question: *I look forward to my future*. Each item is rated on a five-point Likert scale ranging from “completely disagree” (1) to “completely agree” (5). In the present study, its Cronbach α is 0.83, and KMO value is 0.68.

### Chinese version of the brief self control measure (CBSC)

CBSC is the Chinese version of the Brief Self Control Measure developed by Tangney, Baumeister and Boone [42]. It was also adopted by Kim et al. [6]. To guarantee the comparability of these two similar studies, we decided to follow their choice of measure. Moreover, its Chinese version enjoys high reliability for Chinese middle school students (α = 0.81) and college students (α = 0.85) [43]. CBSC consists of 16 items, covering impulsion control, health habits, temptation resistance, concentration, and recreational control. For example, participants might read such a question: *I am good at resisting temptation*. Each item is rated on a five-point Likert scale ranging from “completely not applicable to me” (1) to “completely applicable to me” (5). In the present study, its Cronbach α is 0.86, and KMO value is 0.70.

In addition, we collected participants’ demographic information, such as age, gender, education level, location, and whether they were to have make-up exams for the final exams.

### Data collection and procedure

The survey was launched on 16 January 2023 (the winter holiday started on 9 January 2023) and closed on 22 January 2023. We excluded three participants from our analysis due to inconsistent responses regarding age (they wrote their name or grade instead of age information). Moreover, no outlier was detected because all participants’ scores were within the range of mean score plus or minus three standard divisions. Therefore, the final sample was comprised of 323 participants. More details can be found in Table 1.

### Data analysis

All data were analyzed using IBM SPSS Statistics 25. First, the data were divided into four parts: demographic information, academic procrastination, time attitude, and self-control. Next, Cronbach’s α, mean (M), standard deviation (SD) and correlations (Person Correlation Analysis) were calculated among the four variables. Then, in reference to Li [44], we used Hayes [45] SPSS macro PROCESS (Model 4) (a logistic regression path analysis modeling tool widely used in social sciences for estimating effect in mediator models) based on a 5000 bootstrap to check the mediating role of self-control in the relationship between academic procrastination and time attitude.

## Results

### Demographic information

Table 1 contains the demographic information of all participants. Of them, 178 (55.11%) were female and aged between 12 and 19 years (M = 15.31, SD = 2.89). 175 (54.18%) of them were junior middle school students. They came from three different regions of China: the south-east (133, 41.18%), the centre (67, 20.74%), and the north-west (123, 38.08%). 253 of them (78.33%) said they had to take make-up exams.

**Table 1:** Demographic Information

Age	Range	12–19	
	Mean	15.31 (*SD* = 2.89)	
		Number	Percentage (%)
Gender	Male	145	44.89
	Female	178	55.11
Education level	Junior Middle Schools	175	54.18
	Senior Middle Schools	148	45.82
Location	South-east	133	41.18
	Central	67	20.74
	North-west	123	38.08
Whether make-up exams are needed	Yes	253	78.33
	No	70	21.67

### Descriptive statistics and correlations among variables

Table 2 presents the descriptive statistics and correlations among variables. It shows that academic procrastination was negatively correlated with future positive time attitude (r = − .19, p < .05), present positive attitude (r = − .18, p < .05), and past positive attitude (r = − .14, p < .05), and positively associated with present negative attitude (r = .17, p < .05) at a significant level. Except for the future negative attitude and past negative attitude, our first hypothesis is strongly supported by the results. This is in line with the existing finding that a positive time attitude is negatively correlated with procrastination [6, 31, 34], and a negative time attitude is positively correlated with procrastination [7].

Moreover, self-control negatively correlates with procrastination at a significant level (r = − .53, p < .01), and positively correlates with future positive time attitude (r = .21, p < .05), present positive time attitude (r = .22, p < .05), and past positive time attitude (r = .27, p < .05) at a significant level. This is in line with previous findings that stronger self-control decreases the risk of procrastination [6, 35] and that higher scores on self-control correlate with more optimal emotional responses [42].

**Table 2:** Correlations among variables (CI = 95%)

	M	SD	1	2	3	4	5	6	7	8
1 self-control	3.01	0.49	1							
2 procrastination	2.60	0.43	− 0.53**	1						
3 future positive	2.16	0.77	0.21*	− 0.19*	1					
4 future negative	3.54	0.84	0.16	0.03	− 0.29*	1				
5 present positive	2.53	0.70	0.22*	− 0.18*	0.75**	− 0.11	1			
6 present negative	3.13	0.70	0.22	0.17*	− 0.20	0.75*	− 0.18	1		
7 past positive	2.42	0.67	0.27*	− 0.14*	0.65**	− 0.16	0.60**	− 0.07	1	
8 past negative	3.25	0.87	0.85	− 0.05	− 0.02	0.57**	− 0.03	0.50**	− 0.25*	1

Note: ** means p < .01, * means p < .05

### The mediating effect

SPSS macro PROCESS (Model 4) developed by Hayes [45] was used to check the mediating role of self-control in the relationship between academic procrastination and time attitude (based on a 5000 bootstrap). The results are presented in Table 3. It indicates that self-control significantly mediates the relationship between past positive time attitude and academic procrastination (a*b = − 0.046, 95% CI[-0.069, -0.013], p < .05), and future positive time attitude and academic procrastination (a*b = − 0.037, 95% CI [-0.042, − 0.022], p < .05), and marginally mediates the relationship between present positive time attitude and academic procrastination (a*b = − 0.040, 95% CI [-0.050, − 0.021], p = .06). There is no mediating effect of self-control between all negative time attitudes and academic procrastination. This partially aligns with our second hypothesis that self-control mediates between time attitude and academic procrastination, and is consistent with the findings by Kim et al. [6].

**Table 3:** The Mediating Effect

Path	SC				AP			
	*β*	SE	95% CI		*β*	SE	95% CI	
			LLCI	ULCI			LLCI	ULCI
PAP	0.21*	0.05	0.05	0.27	− 0.05	0.05	− 0.03	0.15
PAN	0.00	0.05	− 0.19	0.01	0.04	0.05	− 0.05	0.14
PP	0.18*	0.04	0.12	0.23	− 0.09*	0.04	− 0.19	− 0.01
PN	− 0.03	0.05	− 0.06	0.11	0.17*	0.05	0.03	0.23
FP	0.17*	0.05	0.08	0.26	− 0.03	0.04	− 0.14	0.06
FN	− 0.01	0.04	− 0.10	0.06	0.03	0.05	− 0.06	0.12
SC					− 0.22*	0.05	− 0.29	− 0.11

Note: PAP = past positive, PAN = past negative, PP = present positive, PN = present negative, FP = future positive, FN = future negative, SC = self-control, AP = academic procrastination, * means p < .05

## Discussion

After the lifting of lockdowns in China, most middle schools postponed the autumn semester’s final exams to the beginning of the following spring semester. That means students had to prepare for the exams during the winter holiday. This unprecedented winter holiday raises the question of whether students would concentrate on preparation, or delay and even avoid doing it during the holiday. Since prior studies identified various associations among academic procrastination, time attitude and self-control, this study goes further to examine the relationship between academic procrastination and all three time attitudes, and the mediating role of self-control in those relationships. We discovered that academic procrastination was negatively correlated with all three positive time attitudes, and positively associated with the present negative attitude. Our first hypothesis is thus supported. We also identified that self-control mediated the relationship between all positive time attitudes and academic procrastination. This certifies our second hypothesis. The explanations and implications of these findings are discussed below, followed by limitations and future prospects.

### The correlation between Time attitude and academic procrastination

Procrastination is a multidimensional construct with time-related factors as the most significant components. The present study found that all positive time attitudes decreased the tendency to procrastinate. This partially supports prior findings that the future-oriented time perspective negatively correlated with procrastination, while the present-oriented time perspective positively correlated with it [6, 8]. One possible reason for this partial support is that we adopted a different measure for time perception. As mentioned, ATAS is designed specifically for adolescents, the age group to which middle school students belong. Thus, we used ATAS in our study, rather than the ZTPI employed by Kim et al. [6] and Taylor and Wilson [8]. Besides, Kim et al. [6] only tested the future and present time perspectives, and Taylor and Wilson [8] only tested the future time perspective. They did not further specify the subdimensions of positive or negative within either future or present time perspective. This may account for our more specific findings as well.

However, our findings agree with the study by Li and Lv [7]. They employed ATAS to investigate the relationship between time attitude and academic procrastination among Chinese middle school students during regular school time. The results showed that all positive time attitudes were negatively correlated with procrastination, and all negative time attitudes were positively associated with procrastination. The fact that the same measure and a similar sample population generated the same results testifies to the rigidity of our results, collected during a special winter holiday. Taken together, either during the semester or a holiday, there is a significant negative correlation between positive time attitude and academic procrastination, and a positive correlation between negative time attitude and academic procrastination among Chinese middle school students. Expectancy-Value Theory [46] might offer an explanation for the results. Researchers in this tradition propose that individuals’ performance can be explained by their expectancies and values, which are, in turn, affected by their affective memories and perceptions of the future. If people value the activities they are involved in and hold a positive attitude toward these activities, they will demonstrate more active performances. In this way, a positive time attitude will surely reduce the risk of academic procrastination. Conversely, a negative time attitude will increase the tendency of academic procrastination.

### The Mediating Role of Self-control between time attitude and academic procrastination

As a stable personality trait, time attitude predicts procrastination behaviour [8, 47]. Yet, the mechanism underlying this correlation is underinvestigated [6, 7]. In the light of self-regulation theory [10], self-control was proposed as the mechanism influencing the relationship between time attitude and procrastination [11]. When making decisions, people who have a positive expectation for future outcomes (time attitude) will regulate their current behaviour (self-control) to seek long-term gratification [48], thus reducing the risk of procrastination. This study offers supportive evidence for this mediating role in addition to the studies by Kim et al. [6]. Students who hold a positive time attitude can appropriately choose beneficial actions in the long run [6], and plan more in order to achieve high self-regulation goals [8]. Naturally, this high self-control plays a vital role in averting procrastination [49]. As a result, self-control displays a significant mediating effect between a positive time attitude and academic procrastination in our study. Thus, it is important not only to foster students’ positive time attitude to overall life, but also to increase their self-control for reducing problematic behaviors. This is because self-control leads people to greater satisfaction, which enhances a positive time attitude. In this way, maladaptive behaviors will be effectively alleviated.

A lack of self-control is a crucial mechanism for procrastination [47, 49]. Generally speaking, people who tend to procrastinate struggle with adaptive emotion regulation and place less value on the rewards of future outcomes. For example, they seldom use adaptive emotion regulation strategies [50], and focus more on short-term mood repair instead of long-term goals [24]. However, people with high self-control implement goal-congruent behaviours by effectively regulating associated negative feelings and modulating the value of each action. They attribute much more value to the benefits of long-term goals, which enables them to be stronger in motivation and execution [51]. Hence, students with higher self-control manifested a positive correlation with positive time attitudes and a lower tendency to procrastinate [6, 20, 47]. This is a reminder for parents and teachers to encourage students’ self-control, so that they can be highly positive and executive in the learning process.

### Implications

Theoretically, the present study, based on self-regulation theory [10], enriches the empirical discussion about the mechanism underlying the correlation between time attitude and academic procrastination. Procrastination is a failure of temporal self-regulation [38]. As an important component of self-regulation [52], self-control may serve as a buffering mechanism between time attitude and academic procrastination, in that students with high self-control can balance the speed of performing a task and the time of completing the task by adjusting and controlling learning behaviours. This results in a low risk of procrastination. The present study provides empirical support for the mechanism with evidence from a different sample population under a different context. The fact that our results agree with those during regular school time [6, 7] guarantees the generalizability of our findings.

Practically, in the face of the prevailing procrastination among students worldwide [3], the present study will be helpful for developing effective interventions. The causal relationship among the three variables suggests that we must not only help students develop a positive attitude toward their time, but also help them learn how to exercise self-control. In addition, since self-control evolves over time [36], it is possible to train students to be more efficient in their self-control, thus enhancing their ability to cope with potential procrastination.

### Limitations

This research has some limitations. Due to the multi-faceted nature of procrastination and self-control, we are reminded of the need to use a more comprehensive methodology to investigate this topic. Nevertheless, both of the two similar studies [6, 7] adopted the survey method, so we decided to be consistent with them. Furthermore, middle schools abruptly announced the postponement of final exams, so this study was initiated hastily to collect data from this novel situation. Therefore, our framework did not include other potential variables, such as academic stress and parental expectations [15, 21, 24]. We hope to design a more comprehensive framework in future studies.

### Future prospects

The current study offers insights into the complex interplay of academic procrastination, time attitude, and self-control during an unconventional winter holiday in China. To comprehensively understand the multi-faceted nature of academic procrastination, future research should extend beyond our focus on time attitude and self-control. Variables such as motivation, self-efficacy, disorganization, time management [3], academic stress, and parental expectations [15, 21, 24] warrant exploration. Investigating these factors would provide a nuanced perspective on the determinants of academic procrastination.

Addressing the challenge of comparability in existing studies, future research should advocate for the standardization of measurement tools for time attitude and procrastination. A common metric would facilitate meta-analyses, fostering a more cohesive understanding of the relationships between time attitude, procrastination, and related variables.

To advance methodological rigor, researchers should consider diverse data collection methods beyond questionnaires. Qualitative interviews, observational analyses, and experimental designs could offer richer insights into the contextual nuances of academic procrastination and its determinants.

Recognizing the dynamic nature of self-control, we recommend incorporating longitudinal designs in future research. Tracking the development of self-control over time would deepen our understanding of its role in academic procrastination and capture dynamic interplays with time attitude and other variables.

In conclusion, future research should broaden its scope by incorporating additional variables, standardizing measurement tools, exploring diverse data collection methods, and adopting longitudinal designs. These endeavors will contribute to a more comprehensive understanding of academic procrastination, informing targeted interventions and educational policies to mitigate its detrimental effects on academic performance.

## Conclusion

Inspired by Self Regulation Theory, this study proposed that self-control plays a mediating role in the relationship between academic procrastination and time attitude, based on the identified correlations among the three variables. Meanwhile, few related empirical studies covered the past time attitude in their frameworks. Thus, this study aimed to fill those gaps. The results of the questionnaire indicated that academic procrastination had a negative correlation with all three positive time attitudes and a positive association with the present negative time attitude. Moreover, self-control significantly mediated the relationship between academic procrastination and all three positive time attitudes. This study reported the correlation between past time attitude and procrastination, compensating for the lack of the past time perspective in the existing research. Moreover, the discovered mediating role of self-control revealed the mechanism by which time attitude affects academic procrastination. Finally, this study contributed to the existing research on this topic with data from a novel context and a different social and cultural background.

### Electronic supplementary material

Below is the link to the electronic supplementary material.

Supplementary Material 1

## Data Availability

The data that support the findings of this study are available from the corresponding author upon reasonable request.
