# Supplementary material for: Time flies, but you’re in control: the mediating effect of self-control between time attitude and academic procrastination
Source: BMC Psychol. 2023 Nov 13;11:393. doi: 10.1186/s40359-023-01438-2 (PMC10644531; doi:10.1186/s40359-023-01438-2)
Supplement: Supplementary file 1 — Supplementary Material 1 [file 40359_2023_1438_MOESM1_ESM.doc]

**Appendix**

**Academic Procrastination Questionnaire for Chinese middle school students (APQC)**

1. I always find myself doing homework that should have been done several days ago.
2. I wait till the last minute to begin doing homework.
3. I wait till the last minute to get up for morning reading.
4. I get up in time for morning reading.
5. Generally, I have fun after I have finished all the homework.
6. Generally, I finish homework a couple of days ahead of the deadline.
7. Even though there is not much homework, I still cannot finish it in the allotted time.
8. I always delay the homework that is necessary to finish.
9. I make decisions with determination.
10. I do not pack my school bag until the last minute before going to school.
11. I am in a hurry to finish my homework on time.
12. Even though it is time to hand in my homework, I am still busy with other things.
13. I delay doing homework when working with classmates.
14. I set about doing homework once it is assigned.
15. I often finish homework in advance.
16. I wait till the last minute to begin doing homework.
17. I wait till the last minute to review, even for an important exam.
18. I often finish all my study plans within a day.
19. I always relax or rest after I have finished all my homework.
20. I keep telling myself: I will finish this homework tomorrow.

**Chinese version of Adolescent Time Attitude Scale (CATAS)**

1. I look forward to my future.
2. I am not satisfied with my life right now.
3. I have very happy memories of my childhood.
4. I doubt I will make something of myself.
5. I am happy with my current life.
6. My past is a time in my life that I would like to forget.
7. My future makes me happy.
8. I have negative feelings about my current situation.
9. I have good memories of growing up.
10. I don’t think I’ll amount to much when I grow up.
11. I am pleased with the present.
12. I am not satisfied with my past.
13. My future makes me smile.
14. I am content with the present.
15. My past makes me sad.
16. Thinking about my future makes me sad.
17. Overall, I feel happy about what I am doing right now.
18. I wish that I did not have the past that I had.
19. I am excited about my future.
20. I am not satisfied with my present.
21. I have happy thoughts about my past.
22. I don’t like to think about my future.
23. I am not happy with my present life.
24. I like to think about my past because it was such a happy time.
25. Thinking ahead is pointless.
26. Overall, I feel happy with my life right now.
27. I have unpleasant thoughts about my past.
28. Thinking about my future excites me.
29. My current life worries me.
30. My past is full of happy memories.

**Chinese version of the Brief Self Control Measure (CBSC)**

1. I am good at resisting temptation.
2. I have a hard time breaking bad habits.
3. I am lazy.
4. I do certain things that are bad for me, if they are fun.
5. People can count on me to keep on schedule.
6. Getting up in the morning is hard for me.
7. People would describe me as impulsive.
8. I spend too much money.
9. I get carried away by my feelings.
10. I do many things on the spur of the moment.
11. People would say that I have iron-clad self-discipline.
12. Pleasure and fun sometimes keep me from getting work done.
13. I have trouble concentrating.
14. I am able to work effectively toward long-term goals.
15. Sometimes, I can’t stop myself from doing something, even if I know it is wrong.
16. I often act without thinking through all the alternatives.
